# Characterization of single-domain antibodies against Foot and Mouth Disease Virus (FMDV) serotype O from a camelid and imaging of FMDV in baby hamster kidney-21 cells with single-domain antibody-quantum dots probes

**DOI:** 10.1186/s12917-015-0437-2

**Published:** 2015-05-22

**Authors:** Di Wang, Shunli Yang, Shuanghui Yin, Youjun Shang, Ping Du, Jianhong Guo, Jijun He, Jianping Cai, Xiangtao Liu

**Affiliations:** State Key Laboratory of Veterinary Etiological Biology, National Foot and Mouth Diseases Reference Laboratory, Lanzhou Veterinary Research Institute, Chinese Academy of Agricultural Sciences, Lanzhou, Gansu China

**Keywords:** Foot-and-mouth disease virus serotype O, Immune library, Single domain antibody, Quantum dots, Probe, Imaging

## Abstract

**Background:**

Foot-and-mouth disease (FMD) is a highly contagious disease that affects cloven-hoofed animals and causes significant economic losses to husbandry worldwide. The variable domain of heavy-chain antibodies (VHHs or single domain antibodies, sdAbs) are single-domain antigen-binding fragments derived from camelid heavy-chain antibodies.

**Results:**

In this work, two sdAbs against FMD virus (FMDV) serotype O were selected from a camelid phage display immune library and expressed in *Escherichia coli*. The serotype specificity and affinity of the sdAbs were identified through enzyme-linked immunosorbent assay and surface plasmon resonance assay. Moreover, the sdAbs were conjugated with quantum dots to constitute probes for imaging FMD virions. Results demonstrated that the two sdAbs were specific for serotype O and shared no cross-reactivity with serotypes A and Asia 1. The equilibrium dissociation constant (KD) values of the two sdAbs ranged from 6.23 nM to 8.24 nM, which indicated high affinity to FMDV antigens. Co-localization with the sdAb-AF488 and sdAb-QD probes indicated the same location of FMDV virions in baby hamster kidney-21 (BHK-21) cells.

**Conclusions:**

sdAb-QD probes are powerful tools to detect and image FMDV in BHK-21 cells.

**Electronic supplementary material:**

The online version of this article (doi:10.1186/s12917-015-0437-2) contains supplementary material, which is available to authorized users.

## Background

Foot-and-mouth disease (FMD), a highly contagious disease from the FMD virus (FMDV), causes significant economic losses on husbandry worldwide. In particular, the 1997 FMD outbreak in Taiwan brought a loss of approximately US$ 1.6 billion to the swine industry [1]. In addition, the 2001 FMDV outbreak in Great Britain caused tremendous losses to the agricultural and tourism industries [2]. Thus, FMD is classified as an International Epizootic Office (OIE) list A disease and is under strict control. FMD is also known as the first animal disease in China. FMDV belongs to the family *Picornaviridae* and mainly infects cloven-hoofed animals. The genome of this virus is a single-stranded RNA (approximately 8.5 kb) that is surrounded by a protein coat consisting of four structure proteins, namely, VP1, VP2, VP3, and VP4 [3]. FMDV is divided into seven immunologically distinct serotypes: A, O, C, Asia I, and South African Territories (SAT1, SAT 2, and SAT3) [4]. These seven serotypes share no cross-immunity. That is, animals that have previously been infected with one serotype remain susceptible to the six other serotypes. Occurring in Europe, South America, and Asia, FMDV serotype O is the most globally prevalent of the seven serotypes.

Immunoglobulins in mammals are commonly assembled from two identical light (L) chains and two identical heavy (H) chains. The N-terminal domains of the H and L polypeptide chains are responsible for antigen recognition. Apart from these conventional antibodies, naturally occurring and functional antibodies that are devoid of light chains are also found in the sera of camelids [5] and a cartilaginous fish [6]. These special antibodies, also known as heavy chain antibodies (HCAbs), lack the first constant domain. The variable domain of the heavy chain from HCAbs (sdAbs or single-domain antibodies) is fully qualified to bind antigens like the Fab fragment (antigen binding fragment) of conventional antibodies. Given their small size, high physicochemical stability, facile genetic manipulation, and capability to bind epitopes inaccessible to conventional antibodies, sdAbs are ideal antigen-binding elements [7]. In addition, sdAbs are highly expressed and easily purified in *Escherichia coli* [8], yeasts [9], mammalian cell lines, and plants [10]. Thus, these antibodies have various medical and biotechnological applications, such as in cancer therapy [11, 12].

**Fig. 1:**
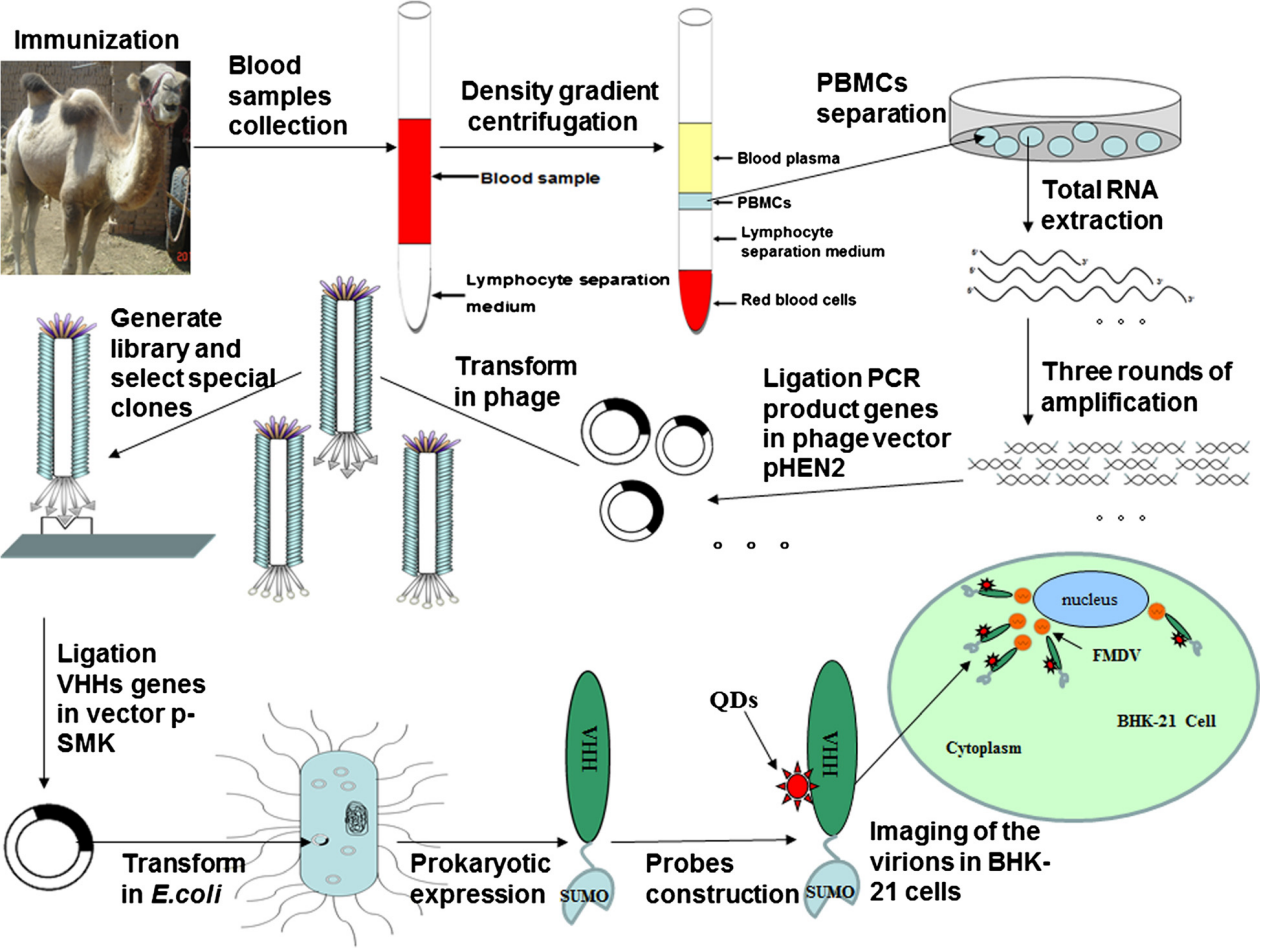
Schematic of strategies for constructing a phage display library and panning of sdAbs against FMDV type O

Fluorescent semi-conductor nanocrystals, also known as quantum dots (QDs), are composed of atoms from elements belonging to groups II to VI (e.g., CdSe, CdTe, CdS, and ZnSe) or III to V (e.g., InP and InAs) in the periodic table. The electronic characteristics of QDs are closely related to its size and shape [13]. Compared with the classical organic dyes, QDs have broader excitation spectra, narrower emission spectra, brighter and more stable fluorescence, and longer lifetimes; thus, QDs can facilitate the long-term bio-imaging of living tissues and cells [14–16]. QD-conjugated antibodies have been used as probes to detect the interaction between viruses and target cells and for the cellular imaging of cancer cells [17, 18]. In the present study, we panned and characterized sdAbs against FMDV serotype O antigens from an immune camelid phage display library. The general construction procedures of a sdAb phage library and the panning procedures of antigen-specific sdAbs are shown in Fig. 1. Results showed that the special sdAbs exhibited a high affinity to FMDV serotype O and shared no cross-reactivity with serotypes A and Asia 1. We also investigated the possibility of constructing sdAb-QD probes for imaging FMDV in BHK-21 cells.

## Methods

### Camelus bactrianus immunization and antibody detection

Two *C. bactrianus* were vaccinated with 2 mL of inactivated FMDV serotype O vaccine (10^7^ 50 % TCID_50_/mL) emulsified in Montanide ISA 206 (ISA) in accordance with previously described methods [18]. The concentration used was based on the weight ratio between porcine and bactrianus camels. Booster immunization was conducted on days 21, 28, 35, and 42. Serum samples were collected after each immunization to monitor the humoral immune response by using a liquid-phase blocking enzyme-linked immunosorbent assay (ELISA). All the FMDV strains, standard sera, and ELISA kit in this work were provided by the OIE/CHINA National Foot and Mouth Disease Reference Laboratory.

All animals were handled in strict accordance with the Animal Ethics Procedures and Guidelines of the People’s Republic of China, and this study was approved by the Animal Ethics Committee of Lanzhou Veterinary Research Institute, Chinese Academy of Agricultural Sciences (No. LVRIAEC2012-006). In this study, the owner of the camels allowed the use of the animals in the experiments. Considerable effort was exerted to minimize suffering.

### Separation of peripheral mononuclear lymphocyte cells

Ficoll–Paque gradient centrifugation is a conventional method to isolate peripheral blood lymphocyte cells (PBLCs) [19]. Seven days after the final inoculation, 100 mL of peripheral blood was collected from the external jugular vein in ethylene diamine tetraacetic acid (EDTA)-coated tubes (50 mL from each camel). The blood samples were mixed with an equal volume of Hank’s buffer and then added into centrifuge tubes that contained appropriate amounts of lymphocyte separation medium. After centrifugation (2000 rpm, 20 min) using a horizontal centrifuge, the target layer was isolated, and the cells in this layer were suspended in minimal essential medium. The cells were incubated at 37 °C in six-well plates to eliminate cell debris. Subsequently, the purified monoclonal cells were divided into aliquots of 6 × 10^8^ PBLCs and then stored in liquid nitrogen.

### SdAb library construction

Total RNA was extracted using RNeasy Plus Mini Kit (Qiagen), and first-strand cDNA was synthesized with oligo-dT primers (Takara). The sdAb fragments were amplified in accordance with previously described procedures [18]. The target fragments that encode HCAbs were amplified with primers LH21 and LH22 (Table 1), which were annealed at both the leader sequences and the CH2 sequences. The fragments (600 bp) from the first amplification were used as templates for the next amplification with primers VHU and VHD, which were annealed at the framework 1 and 4 regions of VHH. The primers VHH*SfiI* and VHH*NotI*, which contain the restriction enzyme cutting sites, were used in the amplification for future steps. The products were digested with restriction enzymes, cloned into the phage vector pHEN2 [20–22] with T4 DNA ligase (New England Biolabs, UK), and then transformed into *E. coli* TG1 cells. The size and diversity of the sdAb library were estimated by plating on LB–ampicillin (100 μg/mL) agar plates, followed by colony PCR with a universal primer pair. The colonies from the plated cells were cultured, subpackaged, and then stored at −80 °C in LB medium supplemented with 50 % glycerine for further use.

**Table 1:** Primers in this work

Primer	Primer sequences (5′–3′)	Restriction site
LH21	GTCCTGGCTGCTCTTCTACAAGG	None
LH22	GGTACGTGCTGTTGAACTGTTCC	None
VHU	GCCATGGCTSAKGTBCAGCTGSTGGAGTCTGG	None
VHD	GCGGCTGAGGAGACGGTGACCWGG	None
VHH*SfiI*	ATTACTGGCCCAGCCGGCCGGCTGAKGTBCAGCTGGTGGAGTCTGGRGGAGGCT	*SfiI*
VHH*NotI*	ATTATTAGTGCGGCCGCTGAGGAGACGGTGACCWGGGTCC	*NotI*
D01U	CTTGGTCTCTGATGTGCAGCTGGTGGAGTC	*BsaI*
D01D	CTCGGATCCTCATGAGGAGACAGTGACCTGG	*BamHI*
A01U	CTTGGTCTCTGATGTGCAGCTGGTGGAGTC	*BsaI*
A01D	CTCGGATCCTCATGAGGAGACGGTGACCTGG	*BamHI*

### Panning of special sdAbs against FMDV type O

In accordance with the principle of phage display [23], the library was infected with M13K07 helper phage and the phage particles were rescued and precipitated with polyethylene glycol. The virus-like particle antigen of FMDV type O was coated overnight at 4 °C at a concentration of 1 mg/mL in carbonate-buffered saline to pan the phages that bind to the antigen-coated immunotubes. The library was subjected to four rounds of panning. To select the VHH antibodies with high affinity, four rounds of panning were performed by increasing the washing time and the concentration of Tween-20 in PBS. The concentrations of Tween-20 were 0.1 %, 0.2 %, 0.3 %, and 0.4 % in the first, second, third, and fourth rounds, respectively. The enriching factors of each panning round were calculated on the basis of the input and output phages. Individual clones that were randomly selected from the four rounds of panning were subjected to monoclonal phage ELISA [24] for detecting the affinity to the target antigen. The plasmids of positive clones were extracted using a plasmid isolation kit (Qiagen Miniprep kit). Local DNA sequencing of unique clones was completed by Sangon Biotech (Shanghai) Co., Ltd.

### Expression and purification of recombinant sdAbs

A p-SMK vector that was fused with a small ubiquitin-like modifier (SUMO) protein tag was employed to promote the expression, solubility, and correct folding of the protein [25]. The presence of a highly stable protein (SUMO) at the N-terminus of a partner protein increases the recombinant fusion protein yield [26]. The sdAb fragments were amplified with primers A01U, A01D, D01U, and D01D, and then cloned into p-SMK vectors after double digestion with restriction enzymes *BsaI* and *BamHI*. The recombinant plasmids were transformed into a BL21-codon-Plus (DE3)-RIL strain (Stratagene), and the cultures were incubated with a shaking culture at 37 °C. The cultures were induced using isopropyl-b-thiogalactopyranoside when the OD_600_ ranged from 0.6 to 0.8. After being incubated in a shaker incubator at 180 rpm for 20 h at 20 °C, the cells were collected through centrifugation and suspended in a 50 mM Tris–HCl buffer (pH 8.0). The recombinant bacteria were ultrasonically treated (three cycles, 10 min/cycle, working for 2 s, and resting for 2 s) to obtain the cell lysates. The proteins of interest were purified using NTA affinity resins (Qiagen) and then identified by SDS–PAGE.

### Identification of recombinant sdAbs through Western blot

After the separation of the protein mixture, Western blot was conducted using the Trans-Blot® TurboTM Transfer System (Bio-Rad, USA). The polyvinylidene fluoride (PVDF) membrane that carried the protein mixture was washed thrice with PBST and blocked with 5 % skim milk at 37 °C for 1 h. Horseradish peroxidase (HRP)-conjugated anti-His monoclonal antibody (diluted 1:1000, Cwbiotech, China) was recognized as the primary antibody. The protein bands were detected with diaminobenzidine reagent.

### Identification of serotype specificity using sandwich ELISA

Sandwich ELISA was used to identify the serotype specificity of the sdAbs. The special sdAbs against FMDV type O were coated in the wells of 96-well microtiter plates to capture antibodies. The plates were incubated overnight at 4 °C. Different viral antigens (inactivated FMDV type A, O, and Asia I, diluted to 16 μg/mL) were added into the corresponding wells. After 1 h of incubation at 37 °C, the wells were washed thrice with PBST. The corresponding serotype-specific rabbit antisera were subsequently added to all wells and incubated for another hour at 37 °C. HRP-conjugated goat anti-rabbit IgG (1:1000) used as the secondary antibody was added into the wells after washing thrice with PBST. 3,3′,5,5′-Tetramethylbenzidine was used as the substrate for the colorimetric reaction, and the reaction was terminated with 2.0 M sulfuric acid. The absorbance was measured at 450 nm with a microplate reader (Bio-Rad). The polyclonal antibodies against FMDV type O and the negative sera from FMDV-free camels were used as the positive and negative controls, respectively.

### Affinity of the sdAbs against FMDV type O

Surface plasmon resonance (SPR) using Biacore 3000 (Biacore, Uppsala, Sweden) was utilized to identify the affinity to specific antigens. The carboxymethyl dextran surface of research-grade CM5 (GE healthcare) chips was activated with a 120 μL mixture (1:1, v/v) of 0.4 M 1-ethyl-3-(3-dimethylaminopropyl) carbodiimide hydrochloride and 0.2 M N-hydroxysuc-cinimide. Recombinant VP1 proteins were immobilized on the surface of reactive CM 5 sensor chips after being diluted to 25 μg/mL to obtain 3000 resonance units using the amine coupling kit. The sdAbs were diluted at concentrations of 25, 50, 75, 100, and 150 nM with HBS-EP buffer and then added onto the sensor chips at a flow rate of 30 μL/min. The binding kinetics were recorded, and the kinetic rate constants (ka and kd) were used to calculate the equilibrium dissociation constant (KD) [27]. Sensor chips were regenerated by 20 mM glycinate hydrochloride (pH 3.0) after each measurement.

### CdSe/ZnS QD-conjugated FMDV type O sdAbs and imaging FMDV in BHK-21 cells

ZnS-capped CdSe ODs (QDs, 605 nm) were synthesized by Wuhan Jiayuan Quantum Dots Co. Ltd. (Wuhan, China). The purified sdAbs were conjugated with QDs in accordance with previously reported procedures [18] to construct two sdAb-QD probes, which were named sdAb-c1-QDs and sdAb-c2-QDs. Alexa Flour 488 (AF488) was used to construct sdAb-AF488 probes (sdAb-c1-AF488 and sdAb-c2-AF488). The conjugation results were determined and visualized using the optimized wide-ranging criteria and agarose gel. For confocal microscopy, a monolayer of baby hamster kidney-21 (BHK-21) cells was cultured in six-well plates and infected with FMDV type O (TCID50 = 10^−6^). After 1.5 h of adsorption, the supernatant was discarded, and fresh Dulbecco’s modified Eagle’s medium supplemented with 2 % fetal bovine serum was added into the wells. The cells were incubated at 37 °C with 5 % CO_2_. At 4 h post infection (h.p.i.), the cells on coverslips were fixed with 4 % paraformaldehyde for 10 min and then permeabilized with 0.5 % TritonX-100 (Sigma, USA) for 20 min at 37 °C. Blocking was performed for 2 h at 37 °C using PBS containing 5 % skim milk powder to reduce unspecific binding. The cells were probed with sdAb-c1-QDs and sdAb-c2-QDs (diluted in PBS) for 1 h at 37 °C. The cells were washed thrice with PBS buffer to terminate the reaction and then processed for the sdAb-AF488 probes to further determine the specificity of the sdAb-QD probes against FMDV in the cells. A 4,6-diamidino-2-phenylindole solution was used to stain the nuclei. The slides were then treated with glycerol jelly mounting medium to prolong storage time. The images were visualized using a laser scanning confocal microscope and saved for further analysis.

## Results

### Construction of sdAb library for FMDV type O

After three rounds of amplification, a 450 bp fragment was obtained, cloned into the phagemid vector pHEN2, and then transformed into *E. coli* TG1 cells. A library named NAL-O was produced. Fifteen randomly selected individual clones were tested for the presence of specific sdAb fragments through PCR. Results demonstrated that all the clones were positive. Local sequencing indicated that each clone contained different complementary determining regions (CDRs). A capacity of 10^8^ was calculated on the basis of the diversity and positive clone number.

**Fig. 2:**
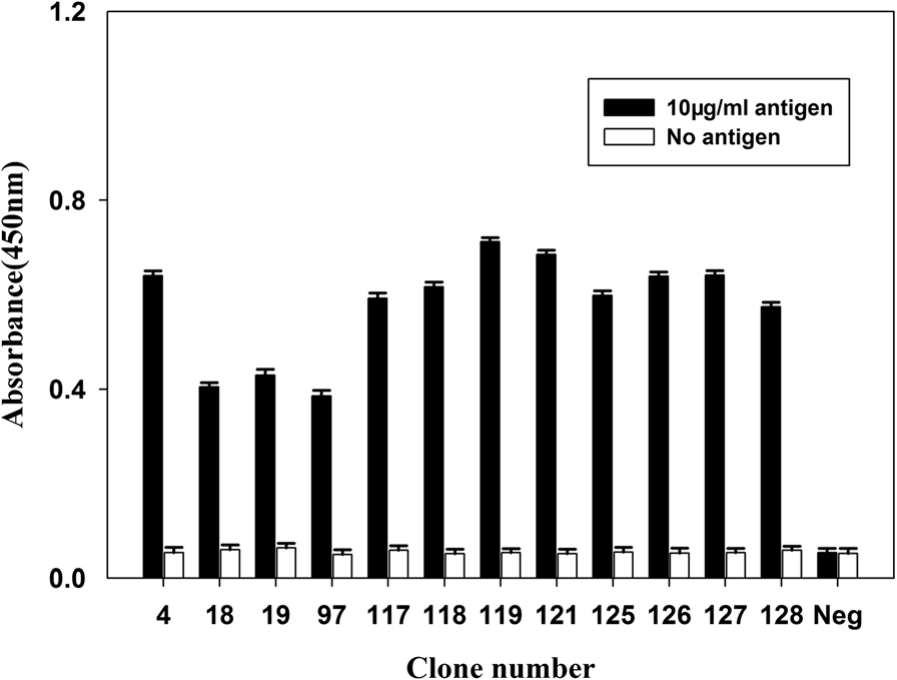
Monoclonal phage ELISA. A total of 131 random clones from the sdAb library were analyzed with monoclonal phage ELISA. FMDV type O antigens at 10 μg/mL were coated in each well. PBS was used as the negative control. A total of 12 clones were selected on the basis of absorbance. The *x-*axis presents the clone number, and the *y*-axis shows the absorbance values at 450 nm

NAL-O was subjected to four rounds of panning for the phage particles against FMDV type O. Phage recovery increased by more than 100-fold after the fourth round of panning than after the first one (Table 2), which demonstrated an efficient enriching factor of antigen-specific sdAbs. A total of 131 individual *E. coli* clones were randomly chosen from NAL-O and analyzed with monoclonal phage ELISA. Twelve clones yielded high absorbance at 450 nm (Fig. 2), and local sequencing of the 12 clones helped identify two sdAb gene sequences, which were named sdAb-c1 and sdAb-c2.

**Table 2:** Enriching factor after each round of panning

Round	Input (^b^pfu)	Output (pfu)	^a^Enriching factor (%)
1^st^	1.0 × 10^11^	1.2 × 10^4^	1.2 × 10^−7^
2^nd^	9.0 × 10^10^	3.2 × 10^4^	3.6 × 10^−7^
3^rd^	8.7 × 10^10^	2.0 × 10^5^	2.3 × 10^−6^
4^th^	2.7 × 10^10^	4.3 × 10^6^	1.6 × 10^−4^

^a^Enriching factors: Enriching factors = output/input. ^b^pfu: plaque-forming unit

**Fig. 3:**
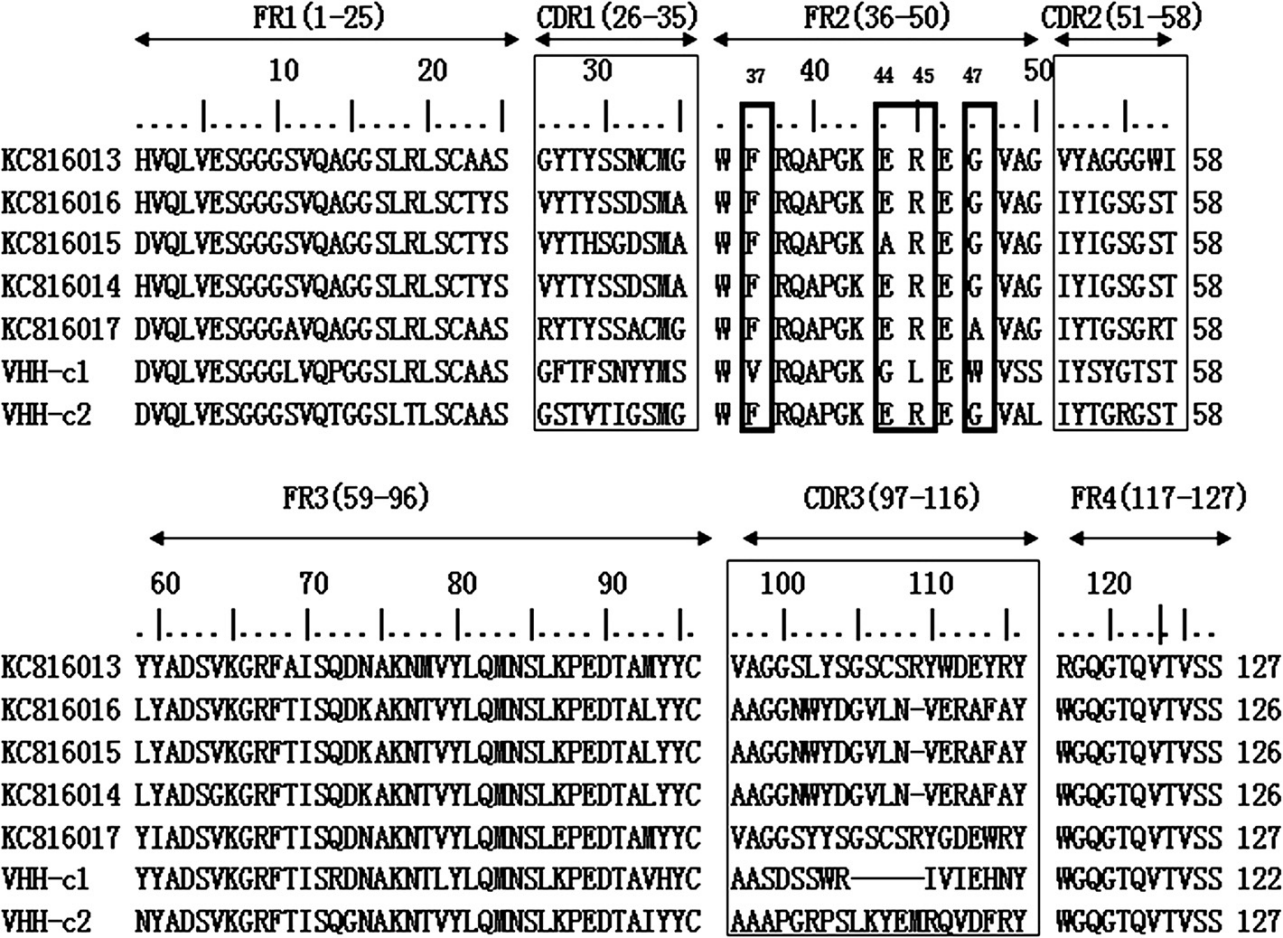
Multiple amino acid sequence alignment of FMDV type O-specific sdAb antibody clones. The framework and CDR regions and amino acid numbering as defined by Kabat are indicated. The CDR regions are boxed in thin lines, and the four conservative hallmark residues of sdAbs in FR2 (Phe37, Glu44Ala, Arg45, and Gly47Ala) are boxed in thick lines. The missing sequences are marked as the dash

The amino acid sequences were subjected to multiple sequence alignment (Fig. 3). Five anti-FMDV Asia 1 sdAbs [GenBank: KC816013, KC816014, KC816015, KC816016, and KC816017] were included in this alignment as references. The alignment results revealed considerable conservation in the framework regions (FR1, FR2, and FR3). The FR2 region in sdAb-c2 contained four hallmark residues (i.e., Phe37, Glu44, Arg45, and Gly47). This indicates that it is derived from a heavy chain antibody [28]. However, sdAb-c1 did not contain these four hallmark residues and was five amino acids shorter than the other sdAbs in the CDR3 region. These findings suggest that sdAb-c1 is a VH-type sdAb.

### Expression and purification of sdAb proteins

**Fig. 4:**
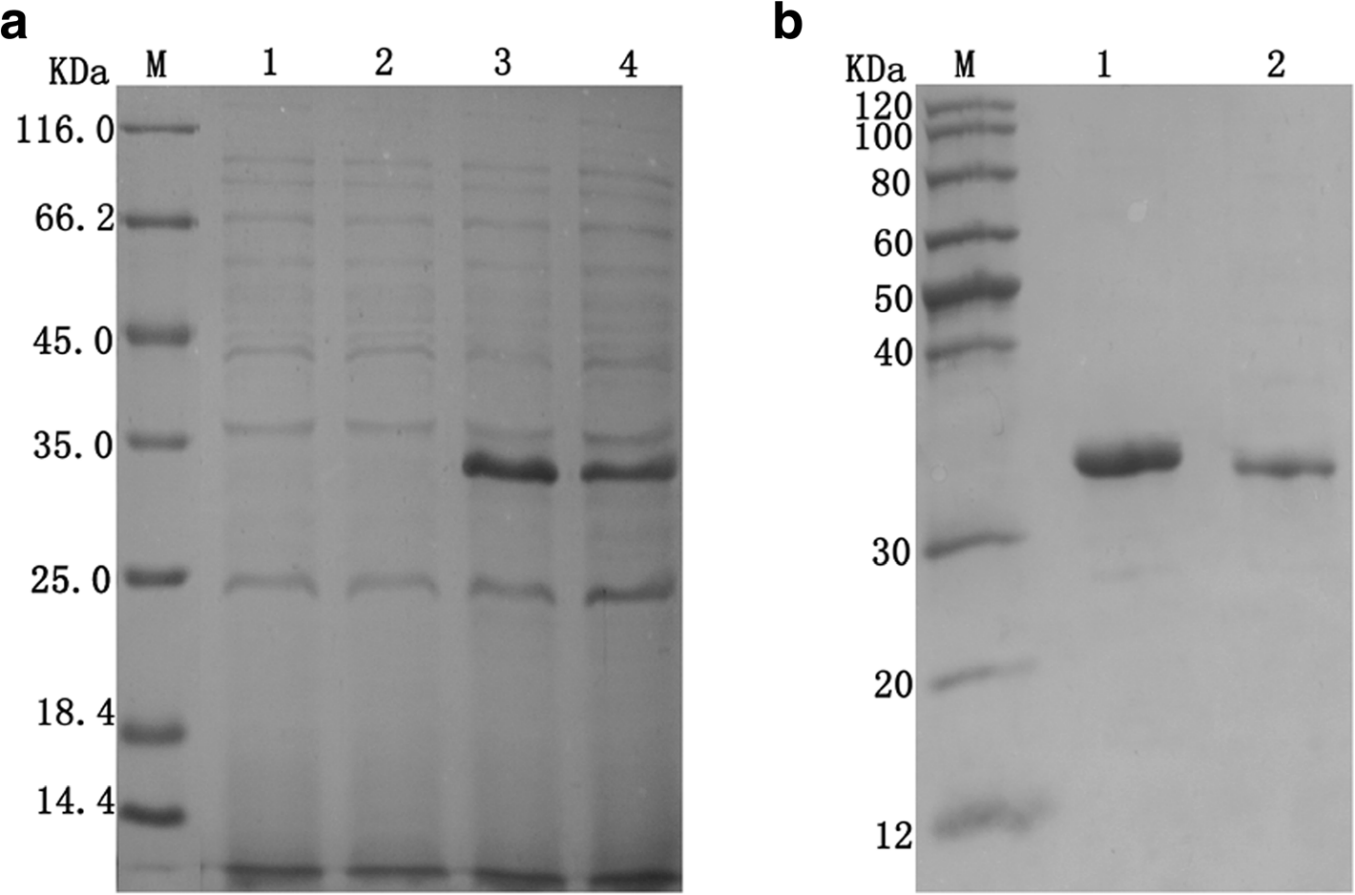
SDS–PAGE analysis of the expression and purification of sdAbs. a Expression of sdAbs in *E. coli*. Lanes 1 and 2, bacterial lysates from the sdAb-c1 and sdAb-c2 groups under non-inducing conditions. Lanes 3 and 4, bacterial lysates from the sdAb-c1 and sdAb-c2 groups under inducing conditions. (b) Purification of recombinant sdAbs protein. Lanes 1 and 2, purified sdAb-c1 and sdAb-c2. M = molecular weight markers, size indicated in kilodalton (kDa)

**Fig. 5:**
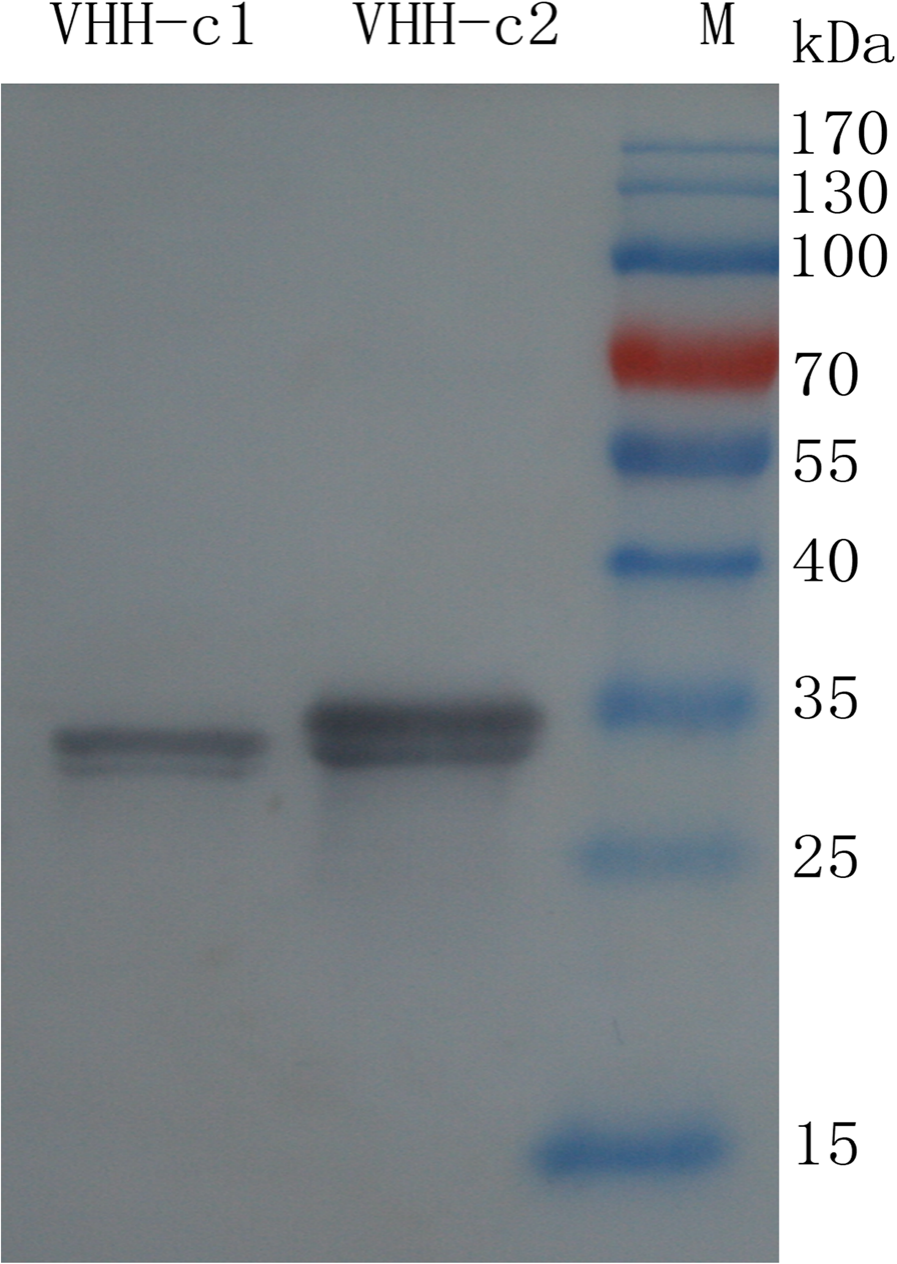
Western blot analysis of the purified sdAbs. SdAbs were expressed through the fusion of 6 × His tag at the N-terminus. Lanes 1 and 2, purified sdAb-c1 and sdAb-c2. M = pre-stained molecular weight markers

The sdAb genes were induced and analyzed using SDS–PAGE (Fig. 4a). The sdAbs had an expected molecular weight of approximately 35 kDa. The purified sdAb recombinant protein was analyzed with a SDS–PAGE gel (Fig. 4b). Only minor nonspecific contaminations were detected in the eluted fraction. The sdAbs were confirmed with Western blot, and the target protein bands (approximately 35 kDa) were observed on the PVDF membrane (Fig. 5).

### Identification of FMDV type O sdAbs

**Fig. 6:**
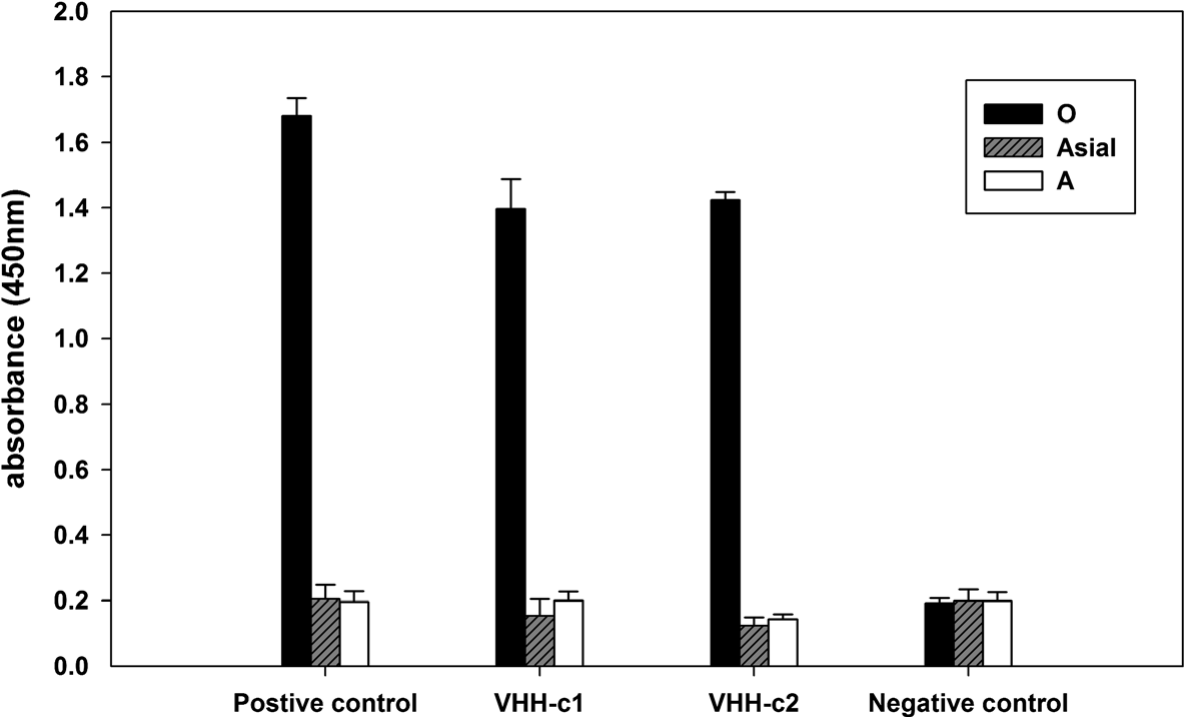
Serotype specificity identification of sdAbs against FMDV serotype O, Asia 1, and A antigens. All samples were tested for absorbance at 450 nm. The negative camel serum was coated as the negative control, and the polyclonal antibodies against FMDV type O were coated as the positive control. Error bars represent the ± standard deviation of three independent measurements

The results of sandwich ELISA showed that the two sdAbs displayed high affinities to FMDV type O and exhibited no cross-reactivity with serotypes Asia 1 and A in ELISA (Fig. 6). The specificities and high affinities of sdAbs to FMDV serotype O indicated that they could be effective antibodies to this target FMDV serotype.

The association and dissociation rate constants curve were generated by biacore evaluation 4.1 software (additional file 1). The SPR results (Table 3) showed that the affinities of sdAbs ranged from 6.23 nM to 8.24 nM, which was within the normal KD range of most sdAbs for their target antigens. These data suggest high affinities to the type O antigen and are consistent with the results of the monoclonal phage ELISA (Fig. 2).

**Table 3:** Kinetics of sdAbs interaction with FMDV antigens

VHH	^a^ka (M^−1^S^−1^)	^b^kd (S^−1^)	^c^KD (M)
A01	1.21 × 10^5^	9.99 × 10^−4^	8.24 × 10^−9^
D01	1.57 × 10^5^	9.75 × 10^−4^	6.23 × 10^−9^

^a^ka : association constant. ^b^kd: dissociation constant

^c^KD: equilibrium dissociation constant, KD(M) = kd(S^−1^) / ka(M^−1^S^−1^)

### Imaging FMDV in BHK-21 cells with sdAb-QD probes

**Fig. 7:**
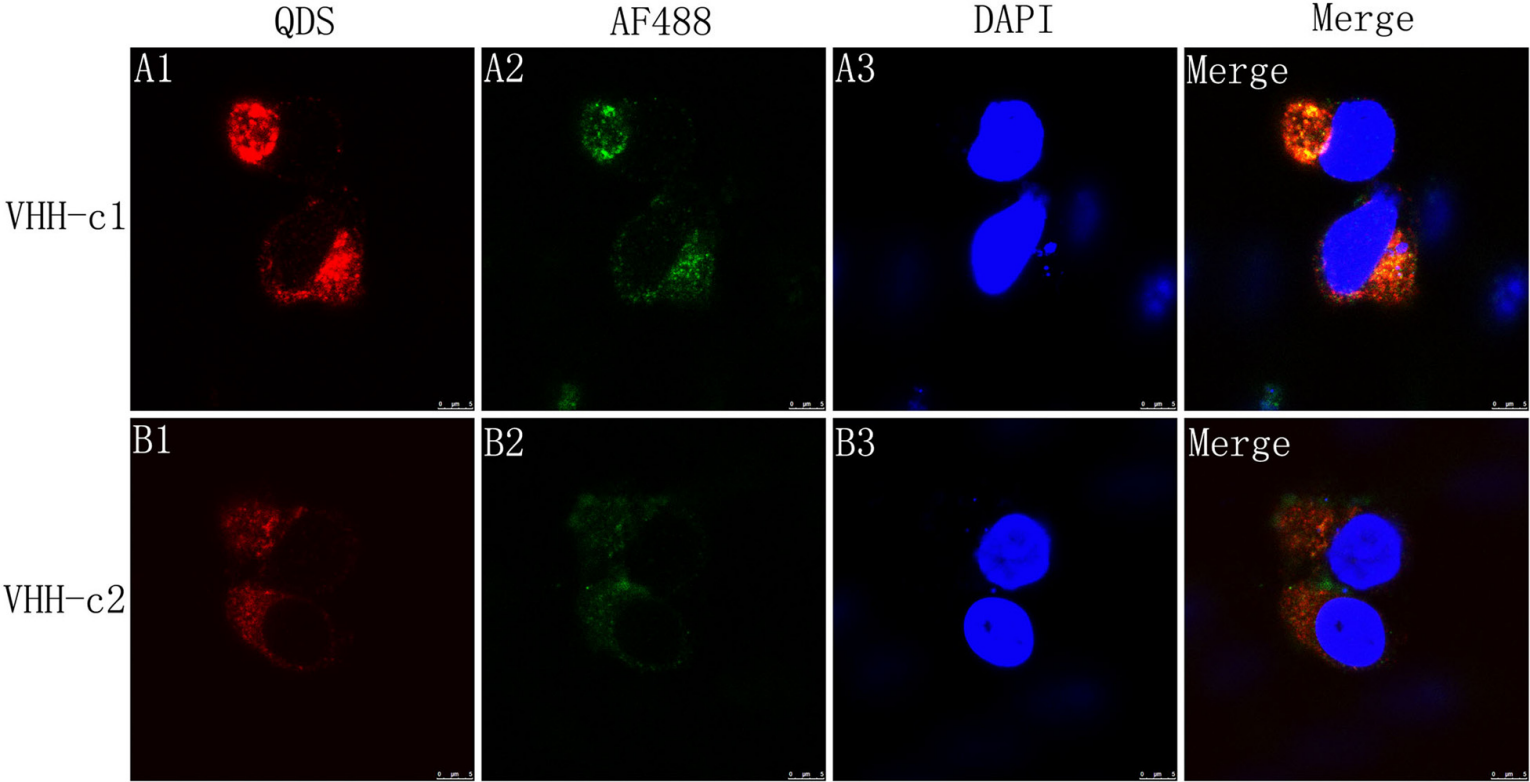
Subcellular localization of FMDV with the sdAb-QD probes. The BHK-21 cells were harvested at 4 h.p.i and processed for immunofluorescence with sdAb-AF488 (green A1 and B1) and sdAb-QDs (red A2 and B2). All nuclei (A3, B3) were stained with DAPI. The merged images show the co-localization of FMDV with different probes. Magnification is 100×

The conjugated products run relatively slow when tested with 1 % agarose gel because of their larger molecular weight than QDs alone (data not shown), indicating that the sdAbs were successfully labeled with QDs. The BHK-21 cells were fixed at 4 h.p.i. and monitored for the presence of FMDV virions using an immunofluorescence assay (IFA). As shown in Fig. 7, the FMDV virions can be observed with sdAb-QD probe staining. To identify the specificity of the sdAb-QD probes *in vitro*, probes sdAb-QDs and sdAb-AF488 were used to stain the fixed cells in a single trial. The merged images in Fig. 7 indicate that FMDV virions were located at the same area as the sdAb-QDs and sdAb-AF488 probes in BHK-21 cells. Most of the virions were located at one side of the nuclei, which indicated a novel invasion mechanism of FMDV when infecting BHK-21 cells. These results suggest that the sdAb-QDs probes are excellent candidates for further research.

## Discussion

Our group previously screened five sdAbs against FMDV type Asia 1 using the immunized library from *C. bactrianus* with Asia 1 virus-like particles. Three of the sdAbs showed high binding affinities and high solubilities. They proved powerful when detecting the virus in host cells. However, FMDV is divided into seven immunologically distinct serotypes that do not share cross-immunity. Serotype O is the most globally prevalent of the seven serotypes of FMDV. Several outbreaks of serotype O have recently occurred in China [29] and neighboring countries [30], suggesting the urgent need to prevent this FMDV serotype. In this work, we constructed a sdAb library for FMDV serotype O and selected the special sdAbs against this serotype. The special sdAbs from this library may serve an important function in the research of serotype O FMDV infection. Antibodies are valuable tools for visualizing antigens in cells because of their high affinity and high specificity [31, 32]. Conventional antibodies and some of their derivatives have been tested as targeting agents, but their development are hindered by disadvantages such as poor stability, aggregation, and high production cost [17]. The use of sdAbs can overcome these disadvantages and enable new functional studies. SdAbs are the smallest functional antibodies and are easily produced in prokaryotic and eukaryotic microorganisms [8, 10, 33]. In addition, they can be widely used in immunofluorescence techniques to capture antigens because of their high affinity [34, 35]. The CDR3 of sdAbs is longer than that of conventional antibodies. Thus, this CDR3 forms a large loop that may help the sdAbs extend in cavities on antigens to bind epitopes inaccessible to conventional antibodies. The sdAbs are also particularly suited for intrabody production because they fold efficiently into functional antigen-binding entities even inside the reducing environment of the cytoplasm [36]. As previously reported [35], sdAbs were fused with fluorescent proteins to generate nanoantibodies that could be expressed in living cells and used to target and trace antigens in live cells. To date, antigens such as protein toxins and poisons, small molecule toxins, and other haptens, viruses, pathogenic bacteria, and parasites are reportedly targeted by sdAbs for diagnosis and therapy [37].

SdAbs can be screened from both immune libraries and non-immune libraries. However, sdAbs derived from non-immune libraries show low affinities for their target antigens [7, 38, 39]. Most immune sdAb libraries are from camels because of their manageability. In this work, two camels devoid of any FMDV vaccine immunization were chosen from the Gobi Desert in Western China. A previous report [40] explained that dromedary camels have low susceptibility to FMDV serotype O and do not transmit FMDV to the susceptible species even through close contact. In addition, high titers of antibodies after the immunization with FMDV antigens make Bactrian camels ideal candidate animals for the production of sdAbs against FMDV. The camels were injected with FMDV type O vaccine to yield an immune library against FMDV. Two unique sdAbs with high affinities to type O FMDV were selected and tested as an efficient tool for targeting specific antigens through phage display technology.

QDs are ideal inorganic luminescent materials that have been widely used for *in vivo* molecular and cellular imaging [41]. In the present work, special sdAbs were conjugated with QDs to generate useful probes for targeting and imaging the FMDV type O virions while infecting BHK-21 cells. BHK-21 cells were fixed at 4 h.p.i., and the FMDV virions were monitored with special probes and a laser scanning confocal microscope. As shown in the images, most of the viruses were clustered at one side of the nuclei. This result is consistent with previous reports [42]. Host cell membranes are rearranged to create vesicular structures when FMDV infects the host cells. Virus genome replication occurs on the vesicular structures. Upon FMDV infection of the cell, cytoplasmic components such as free ribosomes, fragmented rough endoplasmic reticulum, Golgi and smooth membrane-bound vesicles accumulated on one side of the nucleus. The collapse of cellular organelles to one side of the cell was considered as the preparation for virus replication.

## Conclusions

We successfully constructed a specific anti-FMDV type O sdAb immune library using the phage display technology. Two sdAbs from this library were identified through Western blot, ELISA, and SPR. Meanwhile, the two sdAbs were conjugated with QDs to form QD-sdAb probes. The results of the IFA demonstrate that the sdAbs are candidate probes for tracing FMDV type O virions in BHK-21 cells. This study expanded the application of sdAbs as *in vivo* tools for virus infection and replication.

## Additional file

Additional file 1: Figure S1. Binding kinetic measurement. Samples were serial diluted in different concentrations (25, 50, 75, 100 and 150nM). Association and dissociation constants of VHHs were determined separately through SPR measurement using Biacore 3000. The curve shows the result of VHH-c2.

## Abbreviations

FMD: Foot-and-mouth disease
FMDV: Foot-and-mouth disease virus
QD: Quantum dot
sdAb: Single-domain antibody
VHH: The variable domain of the heavy chain from the heavy-chain antibodies
ELISA: Enzyme-linked immunosorbent assay
SPR: Surface plasmon resonance assay
BHK: Baby hamster kidney
