# Supplementary figures and images for: Characterization of single-domain antibodies against Foot and Mouth Disease Virus (FMDV) serotype O from a camelid and imaging of FMDV in baby hamster kidney-21 cells with single-domain antibody-quantum dots probes
Source: BMC Vet Res. 2015 May 22;11:120. doi: 10.1186/s12917-015-0437-2 (PMC4489003; doi:10.1186/s12917-015-0437-2)

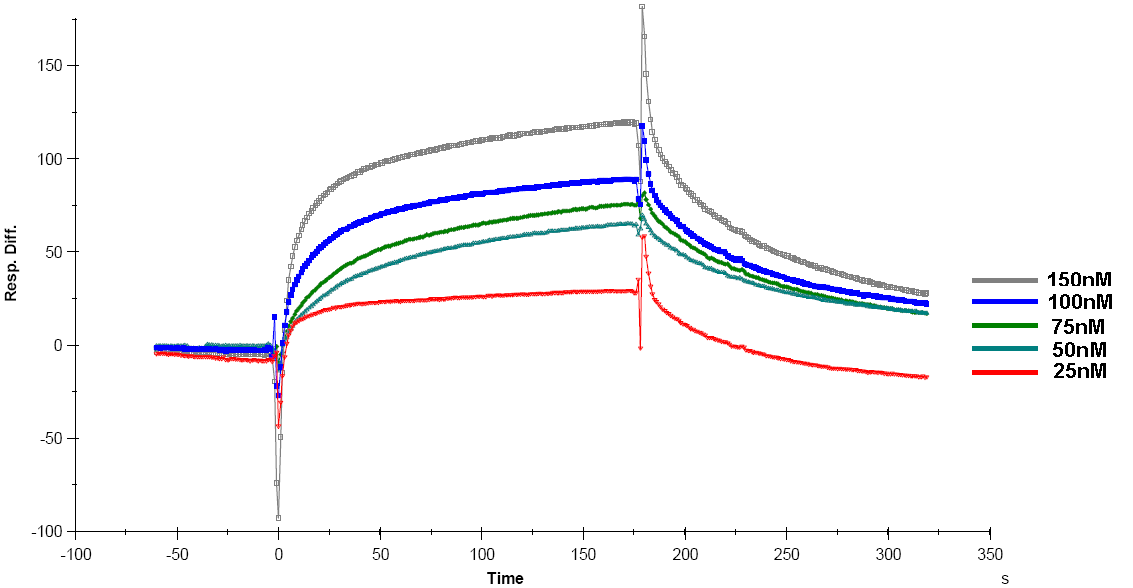

Supplement: Additional file 1: Figure S1. — Binding kinetic measurement. Samples were serial diluted in different concentrations (25, 50, 75, 100 and 150nM). Association and dissociation constants of VHHs were determined separately through SPR measurement using Biacore 3000. The curve shows the result of VHH-c2. [file 12917_2015_437_MOESM1_ESM.tiff]
